# Huntingtin-Interacting Protein 1-Related (HIP1R) Regulates Rheumatoid Arthritis Synovial Fibroblast Invasiveness

**DOI:** 10.3390/cells14070483

**Published:** 2025-03-23

**Authors:** Teresina Laragione, Carolyn Harris, Percio S. Gulko

**Affiliations:** Division of Rheumatology, Department of Medicine, Icahn School of Medicine at Mount Sinai, New York, NY 10029, USA; teresina.laragione@mssm.edu (T.L.); carolyn.harris@mssm.edu (C.H.)

**Keywords:** signaling, fibroblast, rheumatoid, synovitis, synovial, HIP1, HIP1R

## Abstract

Huntingtin-interacting protein 1-related (HIP1R) shares some function similarities with HIP1, and HIP1 regulates arthritis and RA fibroblast-like synoviocytes (FLS) invasiveness. Therefore, we hypothesized that HIP1R might be involved in the regulation of FLS phenotypes and molecular processes relevant to RA. siRNA was used to knockdown HIP1R, HIP1 or control in RA FLS, followed by cell studies for invasion in Matrigel, migration, proliferation, and adhesion. RNA was sequenced and analyzed. HIP1R knockdown significantly reduced RA FLS invasiveness and migration (*p* < 0.05). The DEGs in siRNA HIP1R had an enrichment for GO processes “astrocyte and glial cell projection”, “small GTPase signaling”, and “PDGFR signaling”. The most significantly DEGs had decreased expression in siRNA HIP1R and included AKT1S1, GABBR2, GPR56, and TXNDC12. siRNA HIP1 RA FLS had an enrichment for the “Rap1 signaling pathway” and “Growth factor receptor binding”. The most significantly DEGs in HIP1 siRNA included FGF2, PGF, and SLC39A8. HIP1R and HIP1 DEG lists had a greater than expected number of similar genes (*p* = 0.0015), suggesting that, despite the major differences detected, both have partially overlapping functions in RA FLS. The most significantly DEGs in both HIP1R and HIP1 analyses are involved in cancer cell behaviors and outcomes. HIP1R is a new gene implicated in RA FLS invasiveness and migration, and regulates unique pathways and cell processes relevant to both RA as well as cancer biology. Our study provides new insight into processes implicated in FLS invasiveness, which is relevant for joint damage in RA, and identify new potential gene targets for FLS-specific treatments.

## 1. Introduction

RA is a common autoimmune disease that affects nearly 1% of the population [1,2,3]. RA is associated with chronic pain, joint damage, and increased risk for disability [1,2,3]. Despite the development of new and better treatments over the past two decades, including biologics and JAK inhibitors that target inflammatory mediators and immune cells [4,5,6,7], disease remission remains uncommon. A promising novel strategy might be targeting the fibroblast-like synoviocytes (FLS) [8,9,10].

The RA FLS has a central role in disease pathogenesis and has an altered and invasive phenotype characterized by increased expression of oncogenes and proteases [9,11,12,13,14,15], and is capable of driving an inflammatory and immune response in the joint [16,17]. The in vitro invasive properties of primary cultured RA FLS correlate with radiographic joint damage in patients with RA [18] and with histology damage in rodent models [9]. Furthermore, studies of the invasive properties of FLS have shown promising strategies to identify new and important pathways and genes in disease pathogenesis, and for discovery of new targets for treatments [10,19,20,21,22].

We have recently identified Huntingtin-interacting protein 1 (HIP1) as a new mediator of arthritis severity and joint damage [23]. HIP1 mediates receptor tyrosine kinase (RTK) activation via Rac1 to control FLS invasiveness [23,24]. HIP1 regulates actin cytoskeletal and clathrin-dependent endocytosis, and is also a chaperone for nuclear receptors [25]. HIP1 is expressed in increased levels in some cancers [26,27], and is capable of transforming cells and increasing the accumulation of RTK such as EGFR [28]. We have recently determined that HIP1-binding proteins and HIP1 pathway genes are also involved in the regulation of the RA FLS behavior [29,30].

The Huntingtin-interacting protein 1-related (HIP1R) gene and HIP1 share several protein characteristics and functions. Both HIP1R and HIP1 have TALIN homology domains and can interact with actin [31]. Both also bind inositol lipids via their epsin N-terminal homology (ENTH) domains and regulate clathrin-dependent endocytosis and RTK signaling [31,32,33]. HIP1R has been implicated in cancer cell biology [34,35], though it remains unclear how it contributes to cancer. However, HIP1R has not been studied in the context of RA or RA FLS. Given its similarity to HIP1 functions, we hypothesized that HIP1R might have an important role in RA FLS behavior and on gene pathways and processes implicated in RA. In the present study, we describe the identification of a new role for HIP1R in FLS migration and invasiveness, and describe new pathways and genes it regulates in these cells, including some highly specific for the RA FLS.

## 2. Material and Methods

### 2.1. Isolation and Culture of RA Fibroblast-like Synoviocytes (FLS)

RA FLS were obtained as previously described and under protocol approved by the Icahn School of Medicine at Mount Sinai and the Feinstein Institute institutional review boards (IRB) [20,23]. Briefly, freshly obtained synovial tissues were minced and incubated with a solution containing DNase (0.15 mg/mL), hyaluronidase type I-S (0.15 mg/mL), and collagenase type IA (1 mg/mL) (Sigma) in DMEM (Invitrogen, Carlsbad, CA, USA) for 1 h at 37 °C. Cells were washed and re-suspended in complete media containing DMEM supplemented with 10% fetal bovine serum (FBS; Invitrogen), glutamine (300 ng/mL), amphotericin B (250 μg/mL) (Sigma), and gentamicin (20 μg/mL) (Invitrogen). After overnight culture, non-adherent cells were removed and adherent cells cultured. All experiments were performed with FLS after passage four (>95% CD90+ FLS purity). Nine different RA FLS cell lines, each from a different patient, were used in this study (Table 1).

### 2.2. Immunofluorescence Microscopy

Immunofluorescence was performed as previously reported [20]. Cells were cultured on coverslips overnight in 10% FBS and 10% human serum, then fixed in 4% formaldehyde, permeabilized with PBS-Triton 0.01%, and stained with rabbit anti-HIP1r (AB140608) primary antibody, followed by a secondary florescent antibody Alexa 488 (Invitrogen; 1:100 dilution), phalloidin-594, and/or DAPI-350 (1:100) (Invitrogen). Coverslips were mounted on glass slide and images acquired using a 600× magnification on a LEICA DMi8 microscope (Leica, Buffalo Grove, IL, USA), and analyzed with LASX software, Leica Application Suite X (Leica).

### 2.3. Western Blot

Western blots were done as previously described [23]. Briefly, RA FLS cell lines (*n* = 6, each from a different RA patient) were cultured in 10% FBS and 10% human serum, then lysated in RIPA buffer plus HALT Protease and Phosphatase inhibitor cocktail (78442, Thermo Scientific, Waltham, MA, USA). Total cell lysate (100 μg per lane) was loaded in 4–12% gradient gel (NP0336, Invitrogen, Carlsbad, CA, USA) and transferred to a polyvinylidene fluoride (PVDF) membrane for 1 h. Rabbit HIP1r antibody (AB140608, rabbit, 1:500 dilution) and mouse GAPDH (97166, Cell Signaling, Danvers, MA, USA, mouse, 1:500 dilution) were added to the membrane for 1 h at room temperature followed by secondary fluorescent antibodies goat-anti rabbit IRDye 800 CW (green) and goat-anti mouse 680RD (red) (LI-COR, Lincoln, NE, USA), respectively, for one hour. Images of the bands were capture using Azure Biosystems 600.

### 2.4. siRNA Knock Down

Dharmacon SMARTpool siRNA targeting HIP1R, HIP1, or a non-coding control was purchased from Dharmacon Horizon (Lafayette, CO, USA) and transfected into RA FLS according to the manufacturer’s instructions at a 25 nM concentration. Cells were then incubated at 37 °C for 24–48 h prior to initiating any assay. Knockdown was confirmed by qPCR and RNA sequencing.

### 2.5. Invasion Assay

The in vitro invasiveness of FLS was assayed in a transwell system using Matrigel-coated inserts (BD Biosciences, Franklin Lakes, NJ, USA), as previously described [9,20]. Briefly, 70–80% confluent cells were harvested by trypsin-EDTA digestion, and re-suspended in 500 µL of serum-free DMEM. 2 × 10^4^ cells were placed in the upper compartment of each Matrigel-coated inserts. The lower compartment was filled with media containing 10% FBS and the plates were incubated at 37 °C for 24 h. After 24 h the upper surface of the insert was wiped with cotton swabs to remove non-invading cells and the Matrigel layer. The opposite side of the insert was stained with Crystal Violet (Sigma, Cream Ridge, NJ, USA) and the total number of cells that invaded through Matrigel counted at 25× magnification. Experiments were done in duplicate.

### 2.6. Adhesion to Matrigel

Transfected cells were quickly trypsinized and counted. Six thousand cells per well were plated in triplicate in a 96-well plate previously coated with 5 μg/mL of Matrigel (BD, Franklin Lakes, NJ, USA), in complete media. After two hours, non-adherent cells were washed out with PBS 1× and adherent cells were stained with Crystal Violet. Cells were read with a spectrophotometer at 590 nm.

### 2.7. Migration Assay (Also Called Wound Healing or Scratch Assay)

Transfected FLS were trypsinized and counted. Six thousand cells per well were plated in triplicates in a 96-well plate. The cells were allowed to grow to confluence (usually 24 h). After that, a wound (scratch) was created by using a 5 µL tip. A picture was taken at this time (time 0) and again 24 h later. The migration of cells was determined using imageJ 1.54 g software by subtracting the density (the number of cells that cross into the wound area) after 24 h from the density at time 0 (reference point).

### 2.8. Proliferation

Transfected FLS were trypsinized and counted. Three thousand cells per well were plated in triplicates in a 96-well plate in complete media. After three days, cells were stained with Promega™ CellTiter 96™ AQueous One Solution Cell Proliferation Assay (MTS) (Madison, WI, USA) following the manufacturer instructions. Proliferation was read at 490 nm.

### 2.9. RNA Extraction

FLS from one RA patient was cultured in triplicate to 80% confluence, then transfected with siRNA as described above (three sample replicates per siRNA condition). FLS were then cultured in fresh DMEM media with FBS 10% plus human serum 10% for 24 h, followed by cell collection in RLT buffer with 1% beta-mercaptoethanol. RNA was extracted using RNeasy (Qiagen, Germantown, MD, USA).

### 2.10. RNA Sequencing and Analyses

Total RNA extracted from FLS was quantified by Nanodrop, and 400 ng per cell line per treatment group sent to Novogene (Beijing, China) for sequencing on Illumina platforms, and analyses (for detailed methods see Supplemental Methods File). Briefly, differential expression analysis of two conditions/groups (three biological replicates per condition) was performed using the DESeq2 R package (1.20.0) [36]. The resulting *p*-values were adjusted using Benjamini and Hochberg’s approach for controlling the false discovery rate.

The Disease Ontology (DO) database describes the function of human genes in diseases. DO pathways with corrected *p*-value less than 0.05 were considered significantly enriched by differential expressed genes.

Gene Ontology (GO) enrichment analysis of differentially expressed genes (DEG) was done with the clusterProfiler R package. GO terms (https://www.geneontology.org/) (accessed on 8 March 2024) with corrected *p*-values of less than 0.05 were considered significantly enriched by DEGs. Kyoto Encyclopedia of Genes and Genomes (KEGG) is a database resource for understanding high-level functions and utilities of the biological system, such as the cell, the organism, and the ecosystem, from molecular-level information. KEGG terms (https://www.genome.jp/kegg/) (accessed on 8 March 2024) with a corrected *p*-value less than 0.05 were considered significantly enriched by DEGs.

## 3. Statistics

Means were compared with the *t*-test or paired *t*-test and medians with the rank sum test whenever indicated using GraphPad Prism 6 (San Diego, CA, USA).

## 4. Results

### 4.1. HIP1R Protein Is Present in RA FLS

**Figure 1:**
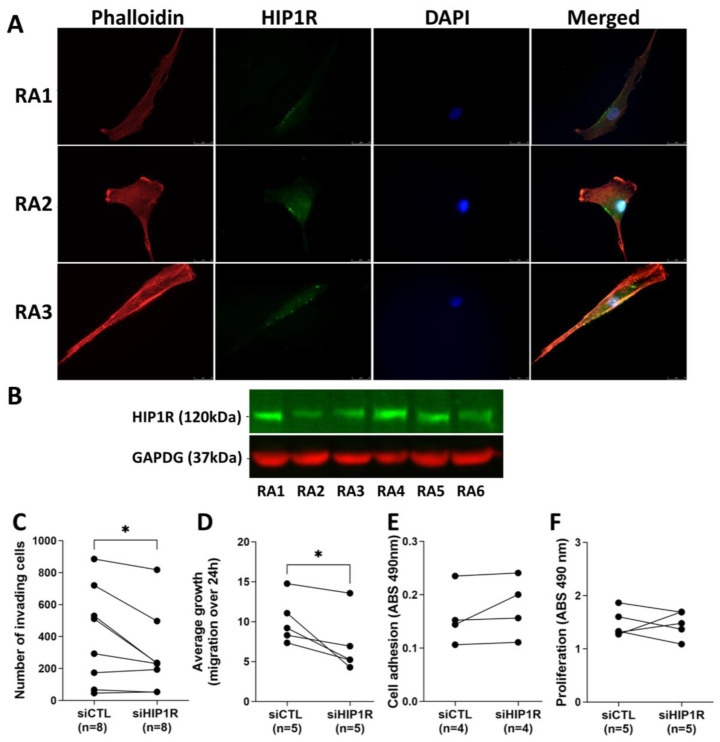
HIP1R protein is present in RA FLS and knockdown significantly affects cell behavior. (**A**) Immunofluorescence staining demonstrating that HIP1R (green) protein is expressed in RA FLS, and localized near the cell edge. Phalloidin stain actin filaments (red) and DAPI stains of the nucleus (blue) (three representative cell lines are shown; 600× magnification). (**B**) Western blots done with RA FLS cell lysates confirming the presence of the HIP1R protein (green) in RA FLS (*n* = 6). GAPDH (red) was used as loading control. (**C**) siRNA knockdown of HIP1R in RA FLS significantly reduced cell invasiveness (*n* = 8, *p* = 0.0419, paired *t*-test), (**D**) and also reduced cell migration in the scratch/wound healing assay (*n* = 5, *p* = 0.0413, paired *t*-test). (**E**) HIP1R knockdown did not have any significant effect in RA FLS adhesion (*n* = 4) (**F**) or on three-day proliferation (*n* = 5). (RA1 to RA6 refers to individual RA patient cell line; ABS = Absorption; see Table 1 for demographic data). (* statistically significant with *p* < 0.05).

Immunofluorescence staining demonstrated that HIP1R protein is expressed in RA FLS (*n* = 6) and localized near the cell edge membrane (Figure 1A). Western blots done with RA FLS cell lysates further confirmed the presence of the HIP1R protein (Figure 1B, *n* = 6).

### 4.2. siRNA Knockdown of HIP1R Significantly Decreased RA FLS Invasiveness and Migration

siRNA knockdown of HIP1R in RA FLS was confirmed by real-time qPCR (Supplemental Figure S1). siRNA knockdown of HIP1R significantly reduced FLS invasiveness (*n* = 8, *p* = 0.0419, paired *t*-test, Figure 1C), and cell migration in the scratch/wound healing assay (*n* = 5, *p* = 0.0413, paired *t*-test; Figure 1D). HIP1R knockdown did not have any significant effect in RA FLS adhesion (Figure 1E) or proliferation (Figure 1F).

**Figure 2:**
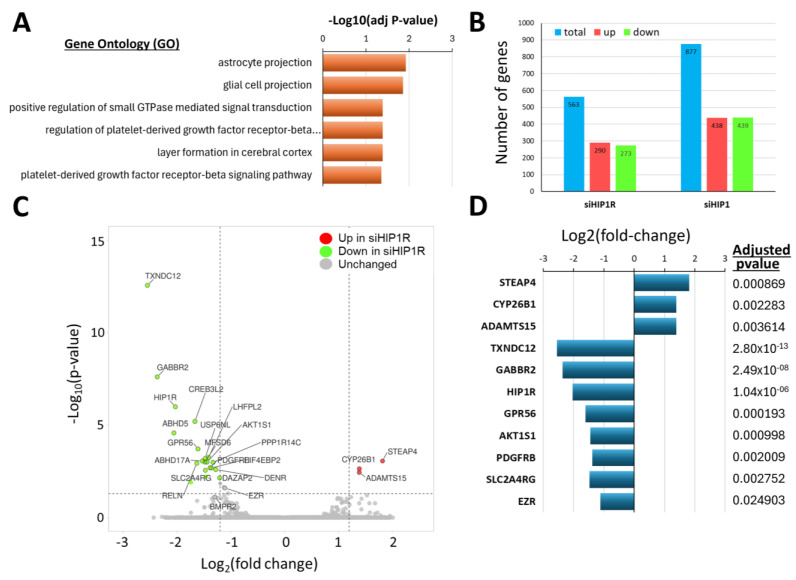
Biologic Pathways and genes enriched in HIP1R knockdown studies. (**A**) GO pathways enriched among DEGs in siRNA HIP1R versus control (adj *p*-value = adjusted *p* value). (**B**) Numbers of DEGs in siRNA HIP1R versus siRNA control, and in siRNA HIP1 versus control. (**C**) Volcano plot showing DEGs (red = up in siRNA HIP1R; green = down in siRNA HIP1R; grey = unchanged). (**D**) DEGs that remained significant after *p*-value adjustment, including down-regulated genes TXNDC12, GABBR2, GPR56, AKT1S1, PDGFRB, HIP1R, SLC2A4RG, and EZR, and up-regulated genes CYP26B1, STEAP4, and ADAMTS15.

Knockdown of HIP1R caused an enrichment of DEGs implicated in RA pathogenic processes, and previously unsuspected astrocyte and glia pathways. To further characterize the pathways and genes regulated by HIP1R, siRNA was used to knockdown this gene in RA FLS. There was an enrichment for DEGs implicated in the GO pathways “Regulation of small GTPase mediated signal transduction”, “Regulation of platelet derived growth factor receptor beta (PDGFRb) signaling”, and “PDGFRb signaling” (Figure 2A and Supplemental Table S1), which have been implicated in RA pathogenesis and in the regulation of RA FLS behaviors, including invasiveness [23,37]. There was also an unexpected enrichment for GO pathways “Astrocyte projection”, “Glial cell projection”, and “Layer formation in cerebral cortex” (Figure 2A and Supplemental Table S1), and those included genes such as ezrin (EZR) and G protein-coupled receptor 56 (GPR56).

### 4.3. Knockdown of HIP1R Was Associated with Unique DEGs Also Involved in Cancer, and in FLS Growth and Invasion

There were 290 genes expressed in increased levels and 273 expressed in lower levels in RA FLS following the knockdown of HIP1R (Figure 2B). The genes with the most significantly decreased expression levels included HIP1R, and genes implicated in cancer cell invasion and proliferation, and in worse cancer outcomes such as thioredoxin domain containing 12 (TXNDC12), gamma-aminobutyric acid type B receptor subunit 2 (GABBR2), GPR56, AKT1 substrate 1 (AKT1S1), PDGFRB, SLC2A4 regulator (SLC2A4RG), and EZR (Figure 2C and Supplemental Table S2). TXNDC12, GABBR2, GPR56, AKT1S1, PDGFRB, SLC2A4RG, and EZR remained significant even after correction of the *p*-values (Figure 2D and Supplemental Table S2).

The genes with the most significantly increased expression in RA FLS knockdown for HIP1R included cytochrome P450 family 26 subfamily B member 1 (CYP26B1), six transmembrane epithelial antigen of prostate 4 metalloreductase (STEAP4), and ADAM metallopeptidase with thrombospondin type 1 motif 15 (ADAMTS15) (Figure 2C), and they remained significant after *p*-value adjustments (Figure 2D; Supplemental Table S2).

Real-time qPCR was used to validate six of the DEG genes in RA FLS cell lines studied in the invasion assays, confirming the reduced expression of PDGFRB, AKT1S1, and HIP1R in the siRNA HIP1R knockdown samples (Supplemental Figure S1). ADAMTS15 and GPR15 trended in the same direction as in the RNA sequencing study, but the difference did not reach statistical significance (Supplemental Figure S1).

### 4.4. Knockdown of HIP1 Caused Enrichment of DEGs Implicated in Different Diseases Such as Cancers and Unique Biologic Processes

HIP1 shares several functions and binding partners with HIP1R. We have previously shown that HIP1 regulates arthritis severity and FLS invasiveness [23], and those discoveries led us to examine other genes in the PDGFR signaling pathway [30] and HIP1 binding partners [29]. Therefore, we examined the effect of siRNA knockdown of HIP1 in RA FLS transcriptomic changes to characterize its target genes and enriched pathways in FLS. We also aimed to determine whether HIP1 and HIP1R shared similar transcriptomic regulatory pathways.

**Figure 3:**
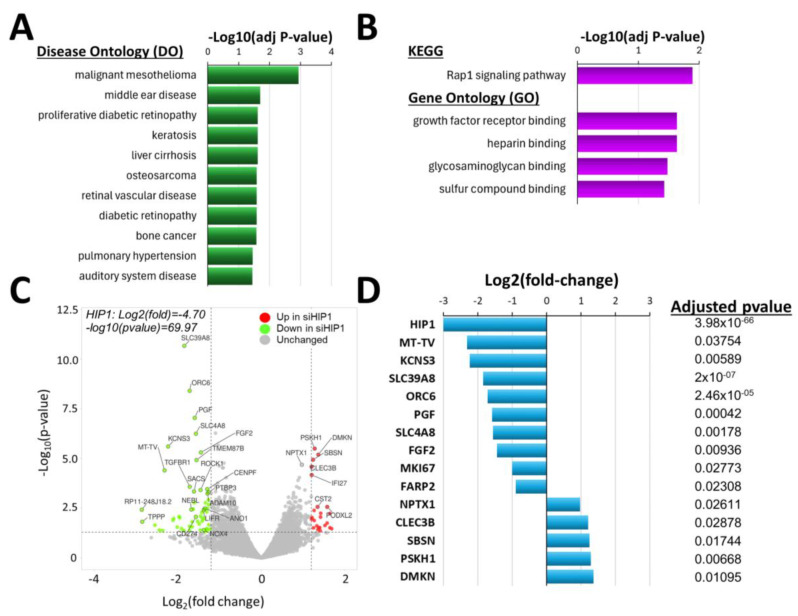
Biologic pathways and genes enriched in HIP1 knockdown studies. (**A**) DO terms such as “malignant mesothelioma”, “proliferative diabetic retinopathy”, “liver cirrhosis”, and “osteosarcoma” were enriched in siRNA HIP1 versus siRNA control analyses (adj *p*-value = adjusted *p* value). (**B**) KEGG pathway “Rap1 signaling pathway” was the most significant, followed by GO pathways “growth factor receptor binding”, “heparin binding”, “glycosaminoglycan binding”, and “sulfur compound binding”. (**C**) Volcano plot showing DEGs (red = up in siRNA HIP1; green = down in siRNA HIP1; grey = unchanged; HIP1 significance and fold change is shown in the upper left corner). (**D**) DEGs that remained significant after *p*-value adjustment for multiple tests included down-regulated FGF2, KCNS3, MT-TV, ORC6, PGF, and SLC39A8, and up-regulated CLEC3B, DMKN, PSKH1, and NPTX1.

HIP1 knockdown was associated with an enrichment of genes implicated in DO terms such as “malignant mesothelioma”, “proliferative diabetic retinopathy”, “liver cirrhosis”, and “osteosarcoma” (Figure 3A and Supplemental Table S3), suggesting that HIP1 controls FLS cellular processes and pathways also relevant to these diseases.

KEGG pathway “Rap1 signaling pathway” was the most significant (Figure 3B; Supplemental Table S4), followed by GO pathways “growth factor receptor binding”, “heparin binding”, “glycosaminoglycan binding”, and “sulfur compound binding” (Figure 3B; Supplemental Table S5). These KEGG and GO pathways included two of the genes with the most significantly reduced expression in HIP1 knockdown cells, PGF and FGF2 (Figure 3C; Supplemental Table S6).

### 4.5. Knockdown of HIP1 Significantly Changed Gene Expression, Including of Some FLS-Specific Genes

There were 439 genes with reduced expression and 438 with increased expression in RA FLS following HIP1 knockdown. The most significantly DEGs had reduced expression following the knockdown of HIP1 and included fibroblast growth factor 2 (FGF2), potassium voltage-gated channel modifier subfamily S member 3 (KCNS3), mitochondrially encoded tRNA valine (MT-TV), origin recognition complex subunit 6 (ORC6), placental growth factor (PGF), Rho-associated coiled-coil containing protein kinase 1 (ROCK1), solute carrier family 39 member 8 (SLC39A8), transforming growth factor beta receptor 1 (TGFBR1), and others (Figure 3C; Supplemental Table S6). Of these genes, FGF2, KCNS3, MT-TV, ORC6, PGF, and SLC39A8 remained significant following the adjustment of the *p*-values (Figure 3D, Supplemental Table S6).

The genes with increased expression in siRNA HIP1 knockdown FLS included C-type lectin domain family 3 member B (CLEC3B), cystatin SA (CST2), dermokine (DMKN), interferon alpha inducible protein 27 (IFI27), podocalyxin-like 2 (PODXL2), protein serine kinase H1 (PSKH1), and neuronal pentraxin 1 (NPTX1) (Figure 3C; Supplemental Table S6). And of these genes, CLEC3B, DMKN, NPTX1, and PSKH1 remained significant after the adjustment of the *p*-value (Figure 3D, Supplemental Table S6). Similar to the observation in the HIP1R studies, the HIP1 DEG had an enrichment for genes implicated in cancer cell behaviors such proliferation and invasion.

### 4.6. Comparison of the DEGs Lists of siRNA HIP1 Versus Control, and of siRNA HIP1R Versus Control

There were fewer DEGs in the HIP1R knockdown analyses (563 DEGs), compared with the HIP1 (877 DEG), suggesting that HIP1 has a greater effect on gene transcriptional signatures. This is in agreement with the known DNA-binding properties and transcriptional regulation associated with HIP1 [38]. We next examined the similarities between these DEG gene lists.

There were 89 DEGs present in both lists, and that number was greater than expected by chance (*p*-value 0.0015, chi-square), suggesting that both have a significant overlap on gene expression, matching their similar effect on FLS invasion. We used Enrichr [39] to analyze the shared list of genes and identified an enrichment for GO processes involving “Negative regulation of Wnt signaling pathway”, “Regulation of macrophage differentiation”, and “Regulation of glial cell differentiation”, suggesting that both are involved in the regulation of these pathways. While these pathways did not remain statistically significant after adjustments of the *p*-value, their identification is relevant to understanding the share role of HIP1R and HIP1 in RA FLS behaviors and in disease pathogenesis (Supplemental Table S7). The most significantly DEGs in the siRNA HIP1R analyses did not overlap with the most significant one in the HIP1 analysis, also suggesting uniqueness in their function.

## 5. Discussion

The behavior of RA FLS resembles that of cancer cells, with increased cell numbers, increased local invasiveness that does not respect tissue boundaries, increased longevity, and increased expression of oncogenes and proteases, among others [9,11,12,13,14,15,40,41]. The in vitro invasive properties of the FLS correlate with radiographic joint damage in patients with RA [18] and with histology damage in rodent models [9], making this in vitro behavior highly relevant to clinical disease. We have previously discovered that HIP1 has a central role in RA FLS invasion and in arthritis severity [23,24]. HIP1 regulates receptor tyrosine kinase (RTK) signaling and the activation of the small GTPase Rac1, and RTK-induced FLS invasiveness [23]. HIP1-binding proteins and HIP1 signaling pathway genes also regulate the RA FLS invasive properties [29,30]. These discoveries provided new insight into the biology of RA FLS, and potentially new targets for treatment.

HIP1R and HIP1 share several functional characteristics and are involved in the regulation of similar cellular processes, such as endocytosis and RTK signaling [31,32,34]. HIP1 has been implicated in different cancers [25,27,31,42], and to a lesser extent so has HIP1R [34]. Therefore, we hypothesized that HIP1R might also regulate RA FLS behaviors, and in this study determined that cells depleted of HIP1R have reduced migration and invasive properties.

RNA sequencing analyses of RA FLS depleted of HIP1R, compared with control, revealed enrichment for GO pathways and processes such as RTK PDGFR pathway signaling, as would be expected given this known function of HIP1R [31,32]. That GO pathway included down-regulated genes PDGFRB and HIP1R. There was also enrichment for “small GTPase signaling”, which to our knowledge has not been previously associated with HIP1R. There were three GO brain-related processes (“astrocyte projections”, “glial cell projection”, and “layer formation in cerebral cortex”) enriched among the HIP1R DEGs, and included three of the most significantly down-regulated genes in HIP1R knockdown FLS, namely GABBR2, GPR56, and EZR.

GABBR2 is one of the receptors for the neurotransmitter GABA [43]. GABBR2 is expressed in the rat synovial tissues [44], but has not been studied in RA. Additionally, its role in inflammation remains unknown. The precise role of GABA in arthritis and inflammation is unclear, with studies suggesting both pro- and anti-inflammatory properties [44,45,46,47,48] perhaps dependent on the binding receptor expressed in the specific target tissue. GPR56 [49] and EZR [50] are implicated in glia and astrocyte biology. EZR expression is increased in highly invasive FLS [15] and it may be involved in RA FLS invasiveness [51]. These observations suggest a new role for HIP1R in the regulation of processes involved in FLS biology, perhaps contributing to local response to neurotransmitters or synovial innervation, as it has in brain cell biology.

The most significant down-regulated gene in siRNA HIP1R was TXNDC12, which promotes cell invasion and metastasis by inhibiting ferroptosis [52]. The other most significant down-regulated genes, including AKT1S1 [53,54], EZR [55,56,57,58], GABBR2 [59,60], GPR56 [61,62,63], HIP1R [34], PDGFRB [64], and SLC2A4RG [65] have also been implicated in cancer cell invasiveness, metastasis, and prognosis. Among these, only EZR [15,51] and PDGFRB [66,67,68,69] were previously implicated in the highly invasive FLS behavior, and in arthritis severity [23,66,67,68,69].

There were fewer genes with a pronounced increased expression in HIP1R knockdown cells. Despite their different biologic functions, the three most significant up-regulated genes, CYP26B1 [70], STEAP4 [71,72], and ADAMTS15 [73], have also been implicated in the regulation of cancer cell biology.

This is not the first time that cancer-related genes are found to be differentially expressed in invasive FLS, or that a parallel between cancer cell behavior and RA FLS is suggested [15,74,75]. However, to our knowledge, this is the first time that HIP1R is implicated in RA FLS behaviors, and we describe a unique and new gene expression signature and cell processes regulated by this gene, and provide new insight into its possible role in joint damage and disease pathogenesis. These observations will also be relevant to cancer.

HIP1 has been implicated in cancer biology [25,26,27,34,76,77], and therefore it was not surprising to identify an enrichment for DO cancers among the DEGs. However, our pathway analyses also identified new processes regulated by HIP1. KEGG process “Rap1 signaling pathway” and GO “growth factor receptor binding”, “heparin binding”, and “glycosaminoglycan binding” were the most significantly enriched processes. The Rap1 gene itself was not among the DEG, but it has been implicated in T-cell mediated protective function in mice with autoimmune arthritis [78] and autoimmune encephalomyelitis [79], and in reducing pro-inflammatory macrophage responses [80]. Rap1 has also been implicated in tumor cell migration and invasion [81]. In our analyses, the KEGG process “Rap1 signaling pathway” included PGF and FGF2, which were two of the most significantly down-regulated genes in HIP1 knockdown cells. PGF is a member of the VEGF family and has angiogenic and pro-inflammatory activity [82]. PGF is expressed in increased levels in RA synovial tissues [83], and increases RA FLS cell proliferation, migration, and invasion [84]. Interestingly, PGF in only expressed by FLS and not by other cells in the RA synovial tissues [16], making it a potentially cell-specific target for treatment. PGF has also been implicated in cancer growth and invasion [85,86,87]. FGF2 has been implicated in cancer [88,89] and has pro-inflammatory activity, including the induction of IL1β and angiogenesis [90,91,92,93].

The genes with the most significantly decreased expression in FLS knockdown for HIP1 included SLC39A8, a zinc and cadmium transporter with pro-inflammatory and arthritis disease severity activity in rodents [94]. SLC39A8 also contributes to neuroblastoma cell growth and metastasis [95]. Another gene significantly down-regulated in HIP1 knockdown cells was ORC6. ORC6 has an essential role in cell replication, and is required for LPS-induced NFkB activation [96], which is a process centrally implicated in RA pathogenesis and joint damage [97,98]. ORC6 also mediates hepatic carcinoma cell invasion [99] and lung cancer progression [100].

The genes with increased expression following HIP1 knockdown included DMKN, which is implicated in melanoma [101], and PSKH1 also implicated in cancer invasion and growth [102,103].

While HIP1R and HIP1 knockdown cells had mostly different DEGs and different enriched pathways and processes, both genes regulate FLS invasiveness and we detected a shared list of DEGs that was greater than expected by chance, underscoring the partial similarities and overlapping of their function. There was also a trend towards enrichment for “Wnt signaling pathway” among that shared list of genes. The Wnt pathway has been implicated in the regulation of the RA FLS function and in disease pathogenesis [104,105,106,107,108]. This is the first time that HIP1R and HIP1 are implicated in the regulation of the Wnt pathway.

## 6. Conclusions

In conclusion, by examining the HIP1 pathway and related genes, we identified HIP1R as a new RA FLS invasion gene. We also report for the first time the DEGs regulated by HIP1R and HIP1 in RA FLS and new pathways and biologic processes, including some FLS specific genes that have the potential to become new targets for treatment. We describe several new genes and biologic processes regulated by HIP1R and HIP1 and anticipate that those findings will be relevant to RA pathogenesis, and perhaps for cancer biology as well, and potentially generate new targets for treatment, including FLS-specific targeting. It will be interesting to expand upon our discoveries to better understand how HIP1R and HIP1 regulated the enriched pathways and DEGs, as well the specific role of these new genes in FLS invasiveness and disease.

## Figures and Tables

**Table 1:** Characteristics of RA patients that donated tissues for development of FLS primary cell lines used this study *.

Donor	Diagnosis	Gender	Ethnicity	Age	Disease Duration (years)	RF	Medication (s)
RA47	RA	Female	Caucasian	66	12	+	Plaquenil (previous MTX use)
RA48	RA	Male	Caucasian	82	20	+	Leflunomide (past Methotrexate)
RA69	RA	M	Caucasian	80	12	−	NSAID, MTX, tofacitinib, simvastatin
RA70	RA	F	Caucasian	69	11	+	Prednisone, MTX
RA72	RA	F	Caucasian	61	22	+	Oxycodone, prednisone
RA73	RA	M	Caucasian	74	32	+	MTX, etanercept
RA2354	RA	F	NA	67	NA	NA	-
RA2383	RA	F	NA	51	NA	NA	Abatacept
RA 2384	RA	NA	NA	NA	NA	NA	-

* MTX: methotrexate; NSAID: non-steroidal anti-inflammatory drug; RF: rheumatoid factor; +: positive; −: negative; NA: not available.

## Data Availability

The RNA sequencing data has been deposited in the NCBI/GEO repository under the accession number GSE278613.

## Supplementary Materials

The following supporting information can be downloaded at: https://www.mdpi.com/article/10.3390/cells14070483/s1, Table S1. Gene Ontology (GO) pathways and processes enriched among the DEGs in siRNA HIP1R vs siRNA control. Table S2. All DEG identified in siRNA HIP1R versus siRNA Control. Table S3. DO enrichment among the DEGs in siRNA HIP1 vs siRNA control. Table S4. KEGG pathways and processes enriched in siRNA HIP1 vs siRNA control. Table S5. Gene Ontology (GO) pathways and processed enriched among the DEGs in siRNA HIP1 vs control. Table S6. All DEGs in siRNA HIP1 vs siRNA control. Table S7. GO biological processes enriched (shared) among DEGs in both siRNA HIP1 and siRNA HIP1R knockdown versus siRNA control. Supplemental Methods. RNA sequencing and analyses detailed methodology. Supplemental Figure S1: qPCR confirmation.
