# Supplementary figures and images for: Huntingtin-Interacting Protein 1-Related (HIP1R) Regulates Rheumatoid Arthritis Synovial Fibroblast Invasiveness
Source: Cells. 2025 Mar 23;14(7):483. doi: 10.3390/cells14070483 (PMC11987873; doi:10.3390/cells14070483)

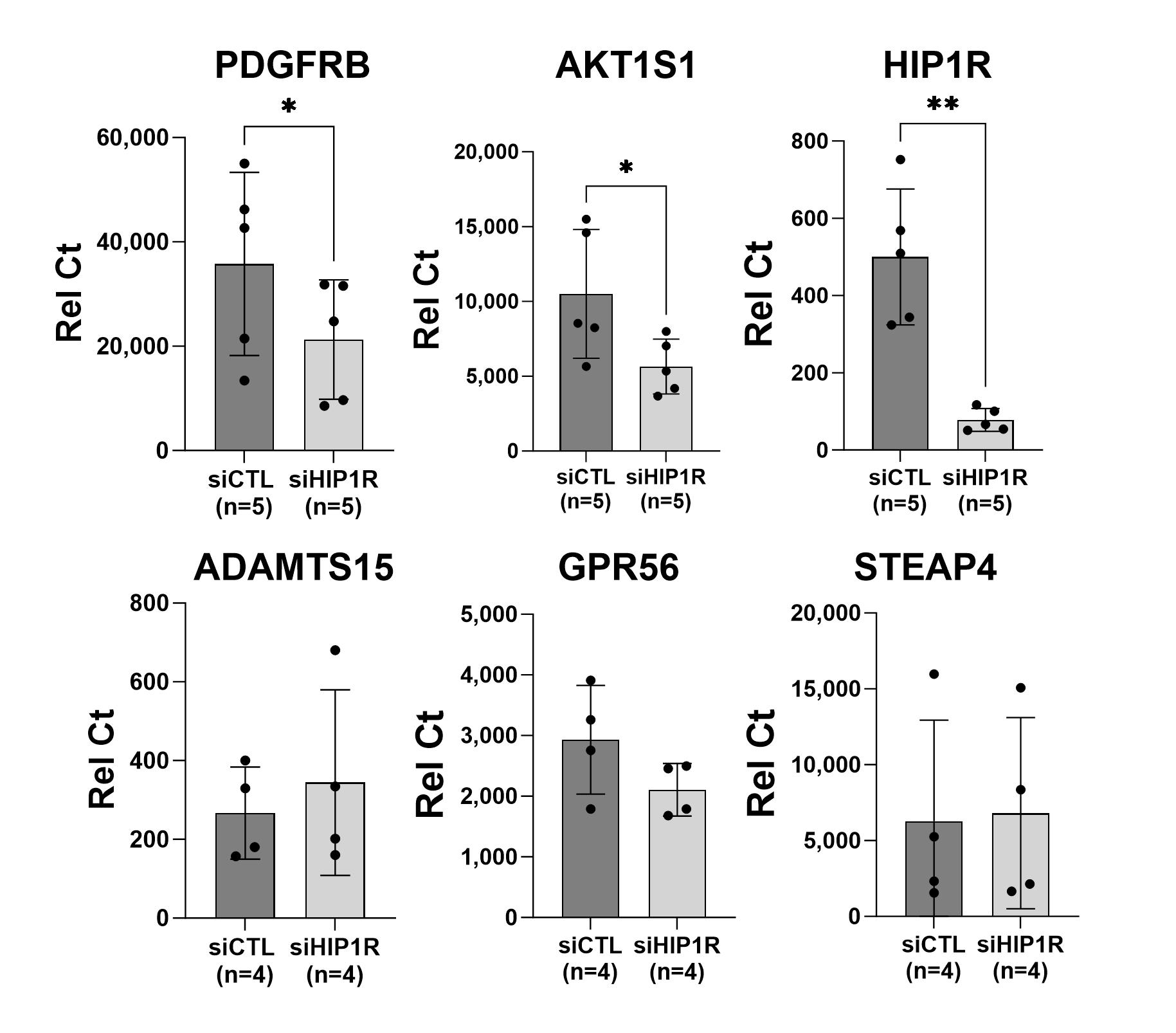

Supplement: Supplementary file 1 [file cells-14-00483-s001.zip › Supplemental Figure S1. qPCR confirmatin.tif]
